# Stabilization and Sterilization of Pericardial Scaffolds by Ultraviolet and Low-Energy Electron Irradiation

**DOI:** 10.1089/ten.tec.2018.0285

**Published:** 2018-12-01

**Authors:** Simona Walker, Jessy Schönfelder, Sems-Malte Tugtekin, Christiane Wetzel, Michael C. Hacker, Michaela Schulz-Siegmund

**Affiliations:** ^1^ Fraunhofer Institute for Organic Electronics, Electron Beam and Plasma Technology FEP, Dresden, Germany.; ^2^ Department of Cardiac Surgery, Faculty of Medicine CGC, Technische Universität Dresden, Herzzentrum Dresden, Dresden, Germany.; ^3^ Institute of Pharmacy, Pharmaceutical Technology, Faculty of Medicine, Leipzig University, Leipzig, Germany.

**Keywords:** pericardium, low-energy electron irradiation, ionizing irradiation, sterilization, glutaraldehyde, SULEEI

## Abstract

Pericardial scaffolds have a wide spectrum of clinical applications ranging from patches for vascular reconstruction and abdominal wall defects to bioprosthetic heart valves. The current gold standard of tissue preparation involves disinfection and cross-linking using glutaraldehyde. However, glutaraldehyde-associated toxicity as well as rapid calcification and premature graft failure represent the major modes of failure.^1^ Therefore, a variety of alternative strategies for tissue conservation have been pursued. However, none of those strategies has substituted glutaraldehyde as the method of choice yet. Furthermore, safe sterilization procedures that are nondetrimental to the tissue's functionality are scarce. We have developed a novel procedure to stabilize and sterilize (S) acellular pericardial scaffolds combining photo-initiated ultraviolet cross-linking (U) with low-energy electron irradiation (LEEI). This SULEEI procedure avoids the use of glutaraldehyde and utilizes LEEI as effective sterilization method. A bioburden of 5.1 × 10^5^ ± 4.6 × 10^5^ viable bacteria could be successfully inactivated by SULEEI treatment applying a surface dose of 30.6 ± 2.8 kGy. By challenging high-density polyethylene foil stacks with >10^6^ *Bacillus pumilus* spores in different depths and modeling the dose distribution within the scaffolds, a maximum sample thickness of 175 μm was determined for successful sterilization. Moreover, SULEEI treatment appeared nondetrimental to the ultimate tensile strength (17.6 ± 8.6 MPa vs. 17.4 ± 9.6 MPa) of the scaffolds compared with glutaraldehyde-treated pericardia. Cell number and overall metabolic activity of human endothelial cells were significantly higher on SULEEI-treated pericardia compared with control samples. In contrast, no cell proliferation could be detected on glutaraldehyde-treated pericardia. Thus, the SULEEI procedure may be a promising novel procedure for glutaraldehyde-free tissue preparation for pericardium-based tissue transplants and tissue engineering.

**Impact Statement:**

Pericardium-based tissue transplantation is a lifesaving treatment. Commercial glutaraldehyde-treated pericardial tissue exhibits cytotoxicity, which is associated with the accelerated graft failure. Replacement of glutaraldehyde has been suggested to overcome those drawbacks. In this study, we report a toxin-free method that combines tissue stabilization with a terminal sterilization. Our data indicate that the SULEEI procedure, which is part of an issued patent, may be a promising first step toward glutaraldehyde-free pericardium-based tissue transplants. Thus, our results may contribute to improving cardiovascular treatment strategies.

## Introduction

Pericardial tissue is in use for a wide spectrum of clinical applications, including patches for vascular reconstruction, abdominal wall defects, and bioprosthetic heart valves.^2–5^ Approximately 600,000 vascular grafts, 20,000,000 patches for abdominal wall repair, and 110,000 bioprosthetic heart valves are transplanted annually worldwide.^6–8^ Since native as well as decellularized xenogeneic pericardia are quickly resorbed *in vivo*,^9,10^ cross-linking with glutaraldehyde is the current gold standard for the conservation of pericardial scaffolds.^11,12^

Despite the cross-linking and disinfecting capacity, cytotoxicity as well as accelerated graft failure due to tissue calcification have been associated with glutaraldehyde treatment.^13^ To overcome those drawbacks, a large number of anticalcification treatments as well as alternative cross-linking strategies have been followed. Compounds that show a lower degree of cytotoxicity including carbodiimides, proanthocyanidin, triglycidylamine, and genipin as well as nontoxic radical scavengers including riboflavin or ascorbate or a combination of various compounds have been evaluated for stabilization of collagen-rich tissues.^14–18^

Sterilization of biological tissues has been challenging. Ideally, a safe sterilization that is nondetrimental to the tissue is warranted. Ionizing irradiation is an established procedure for sterilization of medical products such as surgical instruments according to DIN EN ISO 11137.^19^ During ionizing irradiation, the occurrence of free radicals leads to the eradication of pathogens as well as nonspecific modifications of the substrate.^20^ Low-energy electron irradiation (LEEI) (accelerating voltage of ≤300 kV) has been suggested to be particularly gentle to protein structures. We could recently show that the structure of various virus proteins is almost indistinguishable from that of active pathogens after LEEI treatment.^21,22^ In contrast, high-energy electron irradiation (HEEI) (accelerating voltage of >300 kV) using similar doses as in our study on virus inactivation has been shown to damage tendon allograft structure, which results in decreased mechanical properties.^23,24^ Gamma irradiation has been reported to lead to fibril reorganization, protein denaturation, collagen fragmentation, and condensation of collagen-rich tissues.^14,25–27^ An important advantage of LEEI is its low penetration depth, which is considerably lower compared with HEEI and therefore minimal shielding (in this study: 8 mm of lead) is sufficient for protection against collateral X-rays.^28^ This allows for the integration of LEEI facilities directly at the location of the user such as in hospitals, tissue banks, or good manufacturing practice laboratories.

**FIG. 1.**
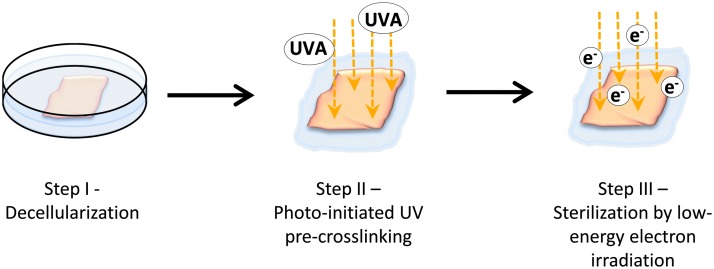
Schematic diagram of the SULEEI procedure. After decellularization of pericardial tissue (step I), pericardia were soaked in riboflavin and UV irradiated (step II) and finally treated with low-energy electron irradiation (step III). UV, ultraviolet; LEEI, low-energy electron irradiation.

Here, we have developed a novel procedure for sterilization (S) and photo-initiated ultraviolet (UV) cross-linking (U) of decellularized pericardial scaffolds combining UV irradiation and LEEI (Fig. 1), the SULEEI procedure. This alternative to glutaraldehyde warranted to overcome tissue cytotoxicity while concurrently improving material properties favorable for pericardium-based tissue transplants. The feasibility of sterilizing pericardial scaffolds by LEEI and the influence of the SULEEI procedure on material properties of the pericardial scaffolds were evaluated by analysis of the enzymatic resistance, the mechanical properties, cytocompatibility, and tissue morphology. Pericardia treated by the SULEEI procedure are referred to as riboflavin/UV–LEEI-treated pericardia.

## Materials and Methods

### Tissue preparation and treatment

Porcine pericardia from 3- to 6-month-old pigs were kindly provided by a local slaughterhouse (Fleischerei Peter, Eschdorf, Germany). Pericardia were obtained under clean conditions, transported to the laboratory in phosphate-buffered saline (PBS) at 4°C, and immediately dissected. Decellularization was carried out as described by Roosens *et al.*^29^ Protocols of the different treatment groups are shown in Table 1. Supple Peri-Guard^®^ (Lamed GmbH, Germany) and Edwards bovine pericardial patch (Edwards Lifesciences Services GmbH, Germany) were kindly provided by the manufacturers. Before analysis, commercial pericardial patches were washed according to the manufacturer's recommendations.

**Table 1.** Protocols Used for the Different Treatment Groups

*Group*	*Treatment*
Native	Native pericardia were stored in PBS at 4°C (control group).
Glutaraldehyde	Pericardia were cross-linked for 30 minutes with 0.2% glutaraldehyde (Applichem, Germany), washed several times, and stored in PBS at 4°C (control group).
dGlutaraldehyde	Pericardia were decellularized as described below and cross-linked with glutaraldehyde as described above (control group).
Decellularized	Pericardia were incubated in 5 mM Tris buffer (pH 8) for 24 h at 4°C. Then, the solution was replaced by fresh 5 mM Tris buffer containing 1% Triton-X-100 (Carl Roth, Germany) for 24 h at 4°C. Next, samples were washed twice for 15 min in HBSS (Carl Roth, Germany) at 4°C. Subsequently, samples were incubated twice with Hanks Balanced Salt Solution (HBSS) containing 100 mg/mL DNAse I (Serva, Germany), 20 mg/mL RNAse A (Serva, Germany), and 100 mg/mL Trypsin (Biochrom, Germany) for 45 min at 37°C. Samples were incubated in fresh 5 mM Tris buffer containing 1% Triton-X-100 for 24 h at 4°C. Samples were washed a few times in PBS at 4°C (according to Roosens *et al.*^29^).
Decellularized–LEEI	Pericardia were decellularized as described above followed by treatment with LEEI.
Riboflavin/UV–LEEI	Pericardial tissue was decellularized as described above and treated with 260 μM riboflavin (Serva, Germany) and 2% dextran T500 (Carl Roth, Germany) for at least 24 h. To prevent drying, samples were placed on filter paper immersed in riboflavin/dextran solution and irradiated with 570 mJ/cm^2^ UVA.
Glucose–LEEI	Pericardia were decellularized as described above, soaked in 10 mM glucose (Merck, Germany) for at least 24 h, and LEEI as described above.
Ascorbate–LEEI	Pericardia were decellularized as described above, soaked in 10 mM ascorbate (Carl Roth, Germany) for at least 24 h, and LEEI as described above.

HBSS, Hank's balanced salt solution; LEEI, low-energy electron irradiation; PBS, phosphate-buffered saline; UV, ultraviolet.

### UV irradiation

Pericardial tissue was treated with 260 μM riboflavin (Serva, Germany) and 2% dextran T500 (Carl Roth, Germany) for at least 24 h. To prevent drying, samples were placed on filter paper immersed in riboflavin/dextran solution and irradiated with 570 mJ/cm^2^ UV (320–480 nm) within 30 min.

### Low-energy electron irradiation

LEEI was carried out for sterilization and modification of pericardial tissue. Pericardia were irradiated in a nitrogen-rich atmosphere (oxygen concentration <100 ppm). Pericardia were wrapped in polyethylene (PE) foil before irradiation for sterility testing or irradiated unpackaged (all other analyses). For LEEI, an accelerating voltage of 150 kV, a beam current of 3 mA, and a working distance of 60 mm were used. The dose was adjusted by adjusting the exposure time to the beam. To obtain a homogeneous dose distribution within the tissue, the pericardia were irradiated from both sides. The applied dose was monitored for every run using the radiochromic Risø B3 film dosimeter (Risø High Dose Reference Laboratory, Denmark) (thickness 18.4 μm, density 1.12 g/cm^3^).^30^

### Quantification of DNA content

Decellularization efficiency was evaluated by quantification of DNA content using the DNeasy Blood & Tissue Kit (Qiagen, Germany) according to the manufacturer's recommendation. DNA content was analyzed using the NanoQuant Plate™ and infinite M200 plate reader (TECAN, Germany). The DNA content was indicated as nanograms of DNA per milligram of the corresponding dry weight.

### Collagenase digestion

Pericardia were incubated in 0.4 U/mL Collagenase NB 8 Broad Range (Serva, Germany) in 50 mM Tris buffer for 3 h at 37°C under constant agitation. Before and after digestion, the pericardia were dabbed four times on an absorbent wipe and the wet weight of the pericardia was determined. The relative weight was calculated by dividing the remaining weight by the initial weight of each sample.

### Determination of the bioburden

For bioburden determination, pericardia were prepared as described above. Instead of irradiation, samples were placed in 3 mL caseine peptone soybean flower peptone bouillon (CASO) and vortexed for 2 min to detach bacteria from the samples. CASO bouillon was plated on CASO agar for viable counts.

The bioburden determination was validated by inoculation of pericardia with 100 colony forming units (CFU) *Bacillus pumilus* spores (MesaLab, France). Bacteria were detached and counted as described above. In parallel, 100 CFU *B. pumilus* were directly inoculated into CASO bouillon. Inoculation was confirmed by viable counts. To compensate for incomplete recovery, the recovery factor was used to determine the overall bioburden $\left( { recovery \ factor = \; { \frac { number \ of \ inoculated \ bacteria }  { number \ of \ recovered \ bacteria } } } \right)$.^19,31^

### Sterility assessment

Irradiated pericardia were carefully unpacked under a laminar flow hood, transferred to sterile CASO bouillon, and incubated at 30°C for 14 days. A clear solution indicated a sterile pericardium, whereas a turbid solution indicated a nonsterile pericardium. As a positive control indicating the absence of inactivating antibiotics, pericardia were inoculated with 100 CFU *B. pumilus* spores (MesaLab, France) and incubated in 3 mL CASO bouillon at 30°C for 14 days. A turbid solution indicated an absent or nonsufficient antimicrobial activity of the pericardium and a successful validation of the sterility assessment.^19,31^

### Simulation of the sterilization depth

A stack of 4, 8, 12, 16, 20, and 26 high-density polyethylene (HDPE) foil disks (Pütz Folien, Germany) (thickness 12 μm, density 0.95 g/cm^3^) was assembled to obtain different thicknesses. The foil in the center of the stack was inoculated with 1.5 × 10^6^ CFU *B. pumilus* spores per square centimeter (MesaLab, France) and dried for 12 h. HDPE foil stacks were packed in PE foil, sealed, and irradiated as described above. After LEEI, samples were carefully unwrapped and transferred to sterile CASO bouillon. Samples were vortexed for 2 min to detach spores from the HDPE foil. CASO bouillon was plated on CASO agar for viability counts. The CASO bouillon–foil suspension was incubated for 14 days at 30°C for sterility assessment as described above. To analyze the dose applied to the *B. pumilus* spores, a stack of 6, 10, 13, and 17 film dosimeters were irradiated from both sides. After irradiation, the stacks were disassembled and the dose that reached each layer was analyzed. From these data, a polynomial regression correlating dose and sample depth was derived (OriginPro 2016). With this regression function, the expected dose at any position within a (tissue) sample was calculated. To confirm the correlation between the penetration depth of the film dosimeter and the pericardial tissue, a sandwich of a film dosimeter surrounded by a pericardium at either side was irradiated from two sides and the dose in the center was recorded.

### Uniaxial tensile test

Uniaxial tensile test was carried out using the Film tester (EZ-Tester; Hegewald & Peschke Mess- und Prüftechnik GmbH, Germany). Rectangular wet pericardial samples (10 × 20 mm) were cut in an apex-to-base direction to compensate for potential direction-dependent effects. Tissue thickness was measured using a foil thickness gauge Model 497 (Erichsen Testing Equipment, Germany). Pericardia were placed on a filter paper and fixed in the clamps. After fixation, the filter paper was removed. Samples were longitudinally elongated at 23°C using a preload of 0.2 N and a strain rate of 4 mm/min. The measurement was stopped at a force reduction of 50%. The stress was calculated by dividing the force by the cross-section. The strain was calculated by dividing the elongation by the initial length of the sample. Young's modulus was calculated by linear regression within the linear region at 10% strain. Ultimate tensile strength and strain at fracture were derived from the data sets.

### Cytocompatibility assessment

Pristine pericardia were disinfected using Polysept^®^ solution (Dermapharm AG, Germany) for 15 min and subsequently washed three times for 10 min with sterile PBS. Pericardia were then placed into well plates to avoid folding of the pericardium. A glass ring (inner diameter of 12.7 mm) was placed on the pericardium to obtain equal surface areas between samples. Human umbilical vein endothelial cells (HUVEC) (PromoCell, Germany) were cultured in endothelial cell growth medium 2 (PromoCell, Germany) supplemented with 10 U/mL penicillin and 100 μg/mL streptomycin at 37°C in a humid atmosphere and 5% CO_2_. Experiments were performed at passage 5. Cells were seeded on the pericardial surfaces at a density of 2.3 × 10^4^ cells per square centimeter. After 48 and 96 h, the supernatant was removed and replaced by fresh medium containing 10% resazurin (Biotinum, Germany). Two hours after a medium change, the fluorescence was measured at an excitation wavelength of 560 nm and an emission wavelength of 690 nm using the infinite M200 plate reader (TECAN, Germany).

Cell number and morphology were assessed using fluorescence microscopy. Cells were washed once with PBS and then fixed with 4% formaldehyde solution (AppliChem, Germany) for 10 min. Thereafter, samples were washed three times with PBS and subsequently permeabilized for 5 min with PBS containing 0.5% Triton-X-100 (Carl Roth, Germany). Samples were washed three times with PBS, and unspecific binding sites were blocked with blocking buffer (2% fetal calf serum [Biochrom, Germany] in PBS). Actin cytoskeleton was stained with 10 μM Phalloidin-TRITC (Sigma–Aldrich, Germany) in blocking buffer for 1 h. After three washing steps with PBS, samples were incubated with 20 μg/mL DAPI (AppliChem, Germany) for 5 min. After removal of the staining solution, samples were mounted with Roti-Mount FluorCare (Carl Roth, Germany). Cell morphology was analyzed using an Olympus BX61 fluorescence microscope. Cell number was determined as the number of DAPI-stained nuclei using the CellProfiler software.

### Histological analysis

Pericardia were fixed in 4% paraformaldehyde (AppliChem, Germany) in PBS, embedded in paraffin, and cut into 4–6 μm thick sections. Decellularization efficiency as well as tissue morphology were assessed using hematoxylin and eosin staining following standard staining protocol. The fiber density was analyzed by quantification of the eosin-stained area of histological cross-sections after hematoxylin and eosin staining and subsequent thresholding using ImageJ 1.46r (National Institute of Health).^32^ Ten randomly selected areas (200 × 80 μm) from three to four different animals and two to three different tissue sections each were used for analysis.

### Scanning electron microscopy

Samples were dehydrated by immersion in ethanol series and dried using critical point drying according to the manufacturer's recommendation (EM CPD300; Leica Leica Methoden Microsystems GmbH). By sputtering, a 6–8 mm thick gold layer was applied. Samples were analyzed in a scanning electron microscope (Zeiss DSM 962) using an acceleration voltage of 15 kV and a working distance of 8 mm.

### Statistical analysis

Statistical analysis was carried out using the GraphPadPrism 6.01 software. Gaussian distribution of the data was analyzed by D'Agostino–Pearson omnibus normality test. To compare two groups that did not follow the Gaussian distribution, the Mann–Whitney test was used. More than two groups that were normally distributed were compared by one-way analysis of variance followed by Bonferroni's multiple comparison test. If more than two groups were compared that failed the normality criterion, a Kruskal–Wallis test followed by Dunn's multiple comparisons test was performed. The mean of a group of measured samples was compared with the calculated value by one sample *t*-test. Values of *p* ≤ 0.05, *p* ≤ 0.01, and *p* ≤ 0.001 were considered statistically significant, depending on the individual experiment.

## Results

### Decellularization

**FIG. 2.**
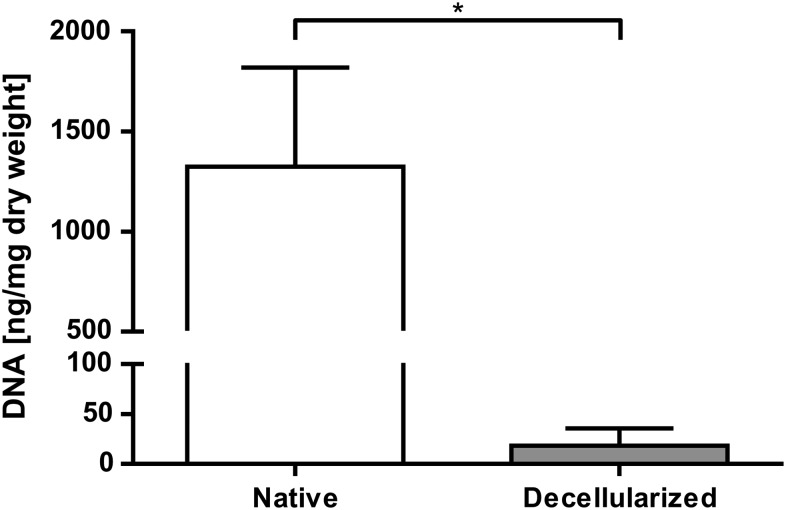
Evaluation of the remaining DNA content after pericardium decellularization. DNA was extracted and the DNA content is shown as nanograms of DNA per milligram of the corresponding dry weight. Data are shown as means + SD (*n* = 9). For statistical analysis, the Mann–Whitney test with a significance level of *p* ≤ 0.001 (*) was applied.

Decellularization efficiency was evaluated by histology as well as analysis of the remaining DNA content. Hematoxylin and eosin staining of decellularized pericardia revealed no nuclear components in any of the analyzed sections (Fig. 7B). Analysis of remaining DNA content showed a 70-fold reduction in DNA content from 1324 ng/mg corresponding dry weight to 18 ng/mg corresponding dry weight (*p* ≤ 0.001) (Fig. 2).

### Biodegradability

**FIG. 3.**
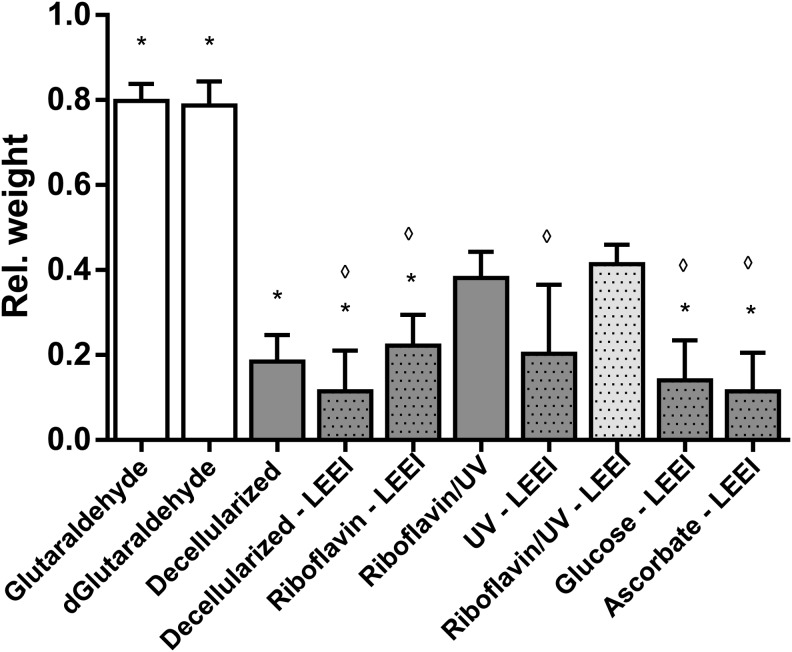
*In vitro* enzymatic resistance of pericardia. Differently treated pericardia (Table 1) were digested for 3 h with 0.4 U/mL collagenase 1. Before and after digestion, the wet weight was determined and indicated relative to the initial weight of each sample. *Dotted bars* indicate LEEI-treated pericardia. Except glutaraldehyde-treated pericardia, all pericardia were decellularized. Data are shown as means + SD (*n* = 9). Statistically significant differences were analyzed by one-way ANOVA followed by Bonferroni's multiple comparisons test. Statistically significant differences (*p ≤ 0.001) of groups compared with riboflavin/UV–LEEI-treated pericardia and statistically significant differences (^◊^*p* ≤ 0.01) of groups compared with riboflavin/UV-treated pericardia were indicated. Differences between glutaraldehyde- as well as dGlutaraldehyde-treated samples and all other treatment groups were statistically significant (*p* ≤ 0.001). No further statistically significant differences were detected. ANOVA, analysis of variance.

Collagenase resistance was analyzed as a measure for *in vivo* biodegradability of pericardia. Glutaraldehyde-treated pericardia lost 20% of their initial weight in the course of 3 h (Fig. 3). In contrast, decellularized pericardia lost 81% of their initial weight. LEEI of samples did not lead to a further weight reduction. Neither the addition of glucose, ascorbate, nor riboflavin alone could increase the remaining weight after 3 h (*p* > 0.05). After cross-linking and sterilization of decellularized pericardia with riboflavin, UV, and LEEI, the collagenase resistance was significantly increased compared with nonirradiated controls (*p* ≤ 0.001). Fifty-nine percent of the tissue was digested after 3 h, whereas more than 80% of tissue was lost in the nonirradiated controls and 20% of the glutaraldehyde-treated tissue (Fig. 3). Due to their improved collagenase resistance, riboflavin/UV and LEEI-treated pericardia were further assessed.

### Sterilization

To estimate the germ reduction by LEEI, the bioburden on riboflavin/UV-treated pericardia was determined. A recovery factor of 1.7 was used for bioburden calculation (*n* = 5, data not shown).^19^ 5.1 × 10^5^ ± 4.6 × 10^5^ CFU/cm^2^ viable bacteria could be detected on riboflavin/UV-treated pericardia (means ± SD, *n* = 20). After LEEI, 100% of pericardia treated with riboflavin/UV became sterile (Table 2). The treatment with glutaraldehyde or riboflavin/UV alone, however, was not sufficient for sterilization (Table 2).

**Table 2.** Sterility Test of Pericardia After Different Treatments

*Treatment*	*Sterile, %*	*Nonsterile, %*	N
Glutaraldehyde	28	72	29
Riboflavin/UV	0	100	30
Riboflavin/UV–LEEI	100	0	30

Samples were incubated for 14 days in caseine peptone soybean flower peptone bouillon at 30°C. A clear solution was considered sterile, whereas a turbid solution was considered nonsterile (*n* = 29–30).

**FIG. 4.**
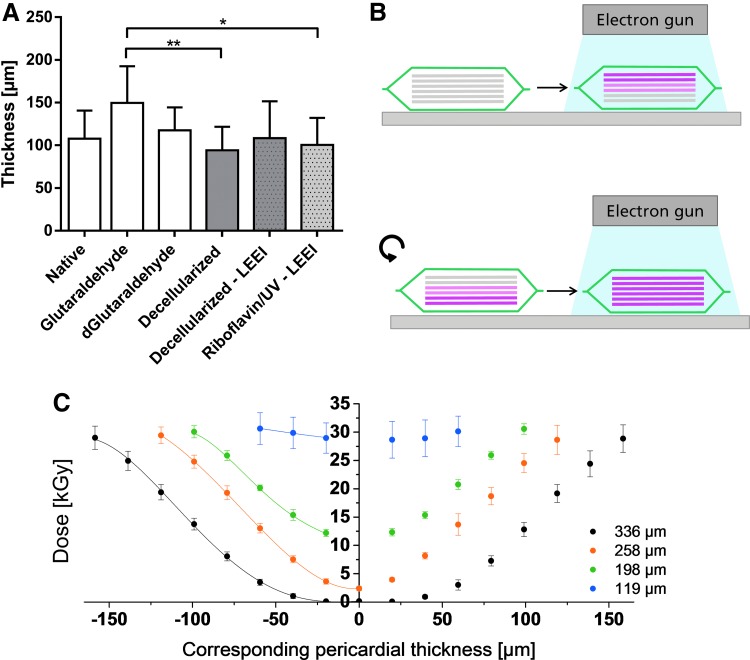
Simulation of dose distribution within the pericardial tissue during LEEI. **(A)** Thickness of differently treated pericardia (Table 1). Data are shown as means + SD (*n* = 9). Statistically significant differences (*p ≤ 0.01 and **p ≤ 0.001) of the indicated groups were determined by the Kruskal–Wallis test followed by Dunn's multiple comparisons test. **(B)** Experimental setup for modelling of the dose distribution within the pericardial tissue. Film dosimeters were assembled horizontally in a stack and irradiated from two sides. After irradiation, the film dosimeter stack was disassembled and the dose of every layer was analyzed. **(C)** Various thicknesses of film dosimeter stacks were irradiated according to **(B)**. Data are shown as means ± SD (*n* = 6). The *lines* indicate regression functions as obtained by polynomial regression for each depth profile.

The penetration depth of electrons into a substrate is mainly determined by the density of the substrate as well as the accelerating voltage used for LEEI.^33^ Since the densities of the different materials used were alike but not equal (density film dosimeter: 1.12 g/cm^3^,^30^ density pericardium: 1.04 g/cm^3^,^34^ density HDPE foil: 0.95 g/cm^3^), all thicknesses specified were adjusted to correspond to the density of pericardial tissue. The mean thickness of riboflavin/UV–LEEI-treated pericardia was 101 ± 32 μm (Fig. 4A). Glutaraldehyde-treated pericardia were significantly thicker (150 ± 43 μm, *p* ≤ 0.01) compared with riboflavin/UV–LEEI-treated pericardia. All other treatment groups did not show significantly different thicknesses compared with riboflavin/UV–LEEI-treated pericardia (Fig. 4A). To model the dose distribution within the pericardial tissue, a stack of six layers of radiochromic film dosimeters with a thickness of 18.4 μm was treated with LEEI from two sides. A mean surface dose of 30.6 ± 2.8 kGy was applied (Fig. 4C). A minimum dose of 28.7 kGy could be maintained throughout the entire sample (Fig. 4C). This dose is above the dose required for ionizing irradiation sterilization by different pharmacopoeia.

Since the penetration depth of LEEI is a critical limitation of this technology, we investigated the maximum thickness of a sample that did just pass the test for sterility, that is, on which no bacterial growth could be detected in the standard test. Various thicknesses of HDPE foil stacks were inoculated on the central layer with 1.5 × 10^6^ CFU *B. pumilus* spores, a reference bioindicator for validation of sterilization processes using ionizing irradiation,^35^ and LEEI was performed from two sides. Stacks up to a thickness of 175 μm could be successfully sterilized. This experiment, however, does not provide information on the sterility assurance level (SAL) at this depth. A stack of 219 μm as well as 285 μm thickness could not be sterilized. A log reduction of 3.3 ± 0.6 and 0.1 ± 0.1 was achieved, respectively, for the central bacterial inoculation (Table 3).

**Table 3.** Simulation of Sterilization Depth

*Corresponding pericardial thickness, μm*	*Inoculum, CFU/cm*^2^	*CFU/cm*^2^*after LEEI*	*Log-reduction*	*Turbidity*	*N*	*Calculated dose, kGy*	*Calculated log-reduction*
44	1.5 × 10^6^	<1	na	−	3	29.7	>6
88	1.5 × 10^6^	<1	na	−	3	28.9	>6
132	1.5 × 10^6^	<1	na	−	3	22.4	>6
175	1.5 × 10^6^	<1	na	−	6	11.1	>6
219	1.5 × 10^6^	1.3 × 10^3^ ± 2.1 × 10^3^	3.3 ± 0.6	+	6	2.5	1.8
285	1.5 × 10^6^	8.1 × 10^5^ ± 5.8 × 10^5^	0.1 ± 0.1	+	6	0.2	0.1

High-density polyethylene foil stacks of the indicated thickness were inoculated with >10^6^ CFU *Bacillus pumilus* spores per square centimeter in the middle and LEEI. Remaining bacteria were determined by viable counts as well as turbidity testing. The dose was calculated by polynomial regression based on the data of Figure 4C. The log reduction was calculated by dividing the dose by the D_10_ value of *B. pumilus* spores. Data are shown as means ± SD (*n* = 3–6). The measured log reduction and the calculated log reduction were compared by one sample *t*-test.

na, not applicable.

To analyze the dose applied on the *B. pumilus* spores within the HDPE foil stack, corresponding thicknesses of film dosimeter stacks were irradiated from both sides and the dose administered on every layer was recorded (Fig. 4B). Doses of 11.2, 2.5, and 0.2 kGy (for the thickness of 175, 219, and 285 μm) were applied to the *B. pumilus* spores (Fig. 4C, Table 3). To confirm that the applied and the calculated doses correspond, a film dosimeter was placed between two pericardia and treated with LEEI from two sides. Subsequently, the applied dose was analyzed and the theoretical dose was calculated by polynomial regression. The mean corresponding thickness of the pericardial sandwich was 193 ± 4 μm (*n* = 6). A dose of 9.5 ± 2.6 kGy (*n* = 6) was determined on the film dosimeter in the middle of the sandwich. The calculated dose accounted 7.8 kGy. The measured dose was not significantly different from the calculated dose (one sample *t*-test, *p* = 0.162).

The D_10_ value describes the dose necessary to reduce a viable population by one power of 10.^36^ A D_10_ value for LEEI of 1.4 kGy was determined for *B. pumilus* by Gotzmann *et al.*^37^ By division of the applied dose by the D_10_ value, the log reduction was calculated. For a thickness of 285 μm, the calculated log reduction and the measured log reduction did not significantly differ (log reduction 0.1 ± 0.1, calculated log reduction 0.1, *p* = 0.59) (Table 3). The calculated log reduction for a thickness of 219 μm was significantly lower compared with the measured log reduction (measured log reduction 3.3 ± 0.6, calculated log reduction 1.8, *p* = 0.002).

### Biomechanical properties

**FIG. 5.**
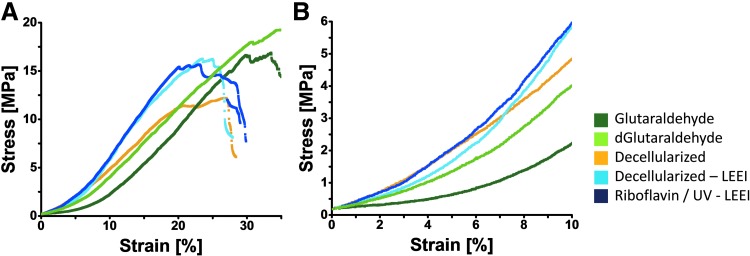
Biomechanical analysis of differently treated pericardia. **(A–B)** Stress–strain curves of differently processed pericardia (Table 1). **(A)** Complete stress–strain curve. **(B)** Magnified view on the physiologically relevant part of the stress–strain curve, that is, 0–10% strain. One representative curve out of *n* = 8 is displayed.

Uniaxial tensile tests were performed to analyze mechanical performance of LEEI pericardia. The stress strain curves are displayed in Figure 5A, B. There were no significant differences between the ultimate tensile strengths of any of the treatment groups (*p* > 0.05) (Table 4). Decellularized as well as nondecellularized glutaraldehyde-treated pericardia showed significantly higher strains at fracture (*p* ≤ 0.001 and *p* ≤ 0.05) and significantly lower Young's moduli (*p* ≤ 0.05) compared with riboflavin/UV–LEEI-treated pericardia. Measuring the stress at 10% strain, riboflavin/UV–LEEI-treated pericardia showed significantly higher stress values compared with glutaraldehyde-cross-linked pericardia (*p* ≤ 0.001 and *p* ≤ 0.01).

**Table 4.** Summary of Biomechanical Properties of Differently Processed Pericardia

*Treatment*	*Young's modulus, MPa*	*Ultimate tensile strength, MPa*	*Strain at fracture, %*	*Stress at 10% strain, MPa*
Glutaraldehyde	0.6 ± 0.3^*^	17.4 ± 9.6	29.5 ± 7.5^*^	2.9 ± 0.9^***^
dGlutaraldehyde	0.6 ± 0.2^*^	17.6 ± 5.5	33.8 ± 10.6^***^	3.4 ± 0.8^**^
Decellularized	0.7 ± 0.4	11.5 ± 4.2	20.2 ± 3.6	4.5 ± 1.6
Decellularized–LEEI	0.9 ± 0.3	15.6 ± 2.9	23.3 ± 4.5	5.6 ± 2.0
Riboflavin/UV–LEEI	1.1 ± 0.4	17.6 ± 8.6	20.1 ± 3.6	6.4 ± 1.8

Data are presented as means ± SD (*n* = 8). Statistically significant differences (^*^p ≤ 0.05, ^**^p ≤ 0.01, and ^***^p ≤ 0.001) of groups compared with riboflavin/UV–LEEI-treated pericardia were analyzed by one-way analysis of variance followed by Bonferroni's multiple comparisons test.

### Cytocompatibility

**FIG. 6.**
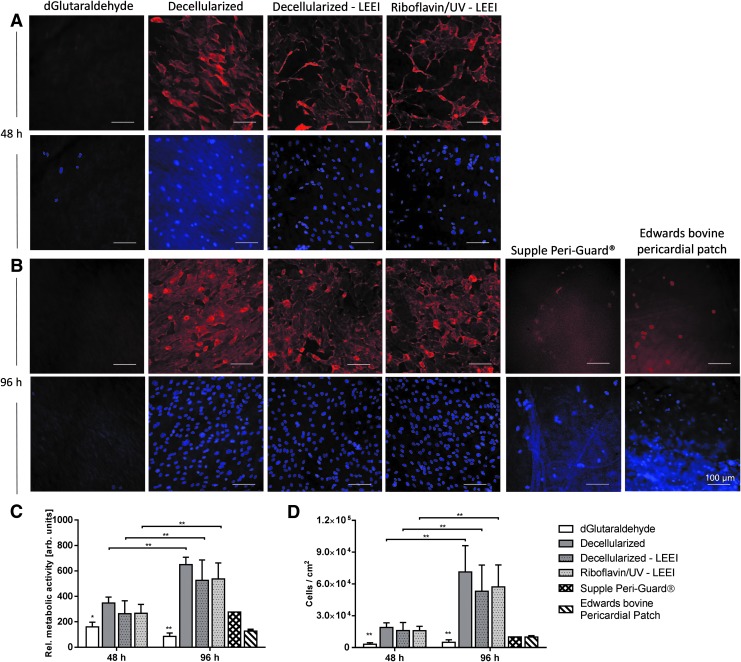
Cytocompatibility of differently processed pericardia. **(A, B)** HUVEC were seeded on differently processed pericardial surfaces (Table 1) for **(A)** 48 h and **(B)** 96 h. Cytoskeleton was stained using Phalloidin. Nuclei were stained using DAPI. Images are representative for *n* = 9 or *n* = 1 (Supple Peri-Guard and Edwards bovine pericardial patch) (scale bar: 100 μm). **(C)** Overall metabolic activity of HUVEC seeded on differently processed pericardia (Table 1) for 48 and 96 h, respectively. **(D)** HUVEC count per square centimeter after 48 or 96 h incubation on pericardia. Nuclei were stained with DAPI and quantified on fluorescence micrographs using CellProfiler software. Data are shown as means + SD (*n* = 9 or *n* = 1 [Supple Peri-Guard and Edwards bovine pericardial patch]). Statistically significant differences (*p ≤ 0.01 and **p ≤ 0.001) of groups compared with riboflavin/UV–LEEI-treated pericardia were analyzed by one-way ANOVA followed by Bonferroni's multiple comparisons test. Differences between dGlutaraldehyde-treated samples and all other treatment groups after 48 h (*p* ≤ 0.05) and 96 h (*p* ≤ 0.001) were statistically significant. No further statistically significant differences were detected. Statistically significant differences (**p ≤ 0.001) of overall metabolic activity and cell number after 48 h compared with after 96 h were analyzed by two-way ANOVA followed by Bonferroni's multiple comparisons test. Supple Peri-Guard^®^ and Edwards bovine pericardial patch were not included into the statistical analysis due to small sample size. HUVEC, human umbilical vein endothelial cells.

To evaluate putative toxicity of riboflavin/UV–LEEI-treated pericardia, cytocompatibility was assessed by seeding HUVEC on the surface of differently treated pericardia and assessing the cell morphology, metabolic activity, and cell number. Fluorescence micrographs revealed a well-spread healthy morphology on riboflavin/UV–LEEI-treated pericardia (Fig. 6A, B). No changes between cells on riboflavin/UV–LEEI-treated pericardia and cells on decellularized pericardia could be detected regarding phenotype (Fig. 6A, B).

The relative overall metabolic activity of HUVEC on glutaraldehyde-treated pericardia was significantly lower compared with HUVEC on riboflavin/UV–LEEI-treated pericardia after 48 and 96 h (*p* ≤ 0.01 and *p* ≤ 0.001) (Fig. 6C). Additionally, the overall metabolic activity was significantly increased after 96 h compared with 48 h in all treatment groups except the glutaraldehyde group (*p* ≤ 0.001) (Fig. 6C). Quantification of the cell number revealed similar results (Fig. 6D).

Each manufacturer has an individual preservation regimen for pericardial patches and heart valves, including glutaraldehyde concentrations, washing regimens, and anticalcification post-treatments. Since those factors influence cell adhesion and viability on the tissue, two commercially available pericardial patches were analyzed. Ninety-six hours after HUVEC seeding, cells were rounded up on Supple Peri-Guard and Edwards bovine pericardial patch (Fig. 6B). The overall metabolic activity and the cell number of HUVEC on both Supple Peri-Guard and Edwards bovine pericardial patch were considerably lower compared with riboflavin/UV–LEEI-treated pericardia (Fig. 6C, D).

### Tissue morphology

**FIG. 7.**
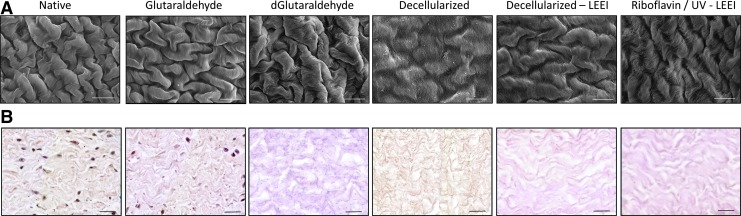
Morphological analysis of differently processed pericardia. **(A)** Scanning electron micrographs (scale bar: 20 μm) and **(B)** hematoxylin and eosin staining (scale bar: 20 μm) of differently processed pericardia (Table 1). Images are representative for *n* = 3.

To assess the morphological changes in the tissue architecture caused by the different treatments, tissue surface microstructure was analyzed by scanning electron microscopy. A qualitative assessment of tissue morphology of the different treatment groups is summarized in Supplementary Table S1 (Supplementary Data are available online at www.liebertpub.com/tec). Native pericardia showed a network of randomly oriented collagen fibers. The collagen network of decellularized pericardia was of a looser and more swollen morphology (Fig. 7A). In hematoxylin and eosin-stained tissue sections, fiber density was analyzed by quantification of the eosin-positive area fraction (Table 5). Glutaraldehyde-treated pericardia showed a tendency toward higher fiber densities compared with all other treatment groups (Table 5, Fig. 7B).

**Table 5.** Quantification of the Fiber Density of Differently Treated Pericardia

*Treatment*	*Native*	*Glutaraldehyde*	*dGlutaraldehyde*	*Decellularized*	*Decellularized–LEEI*	*Riboflavin/UV–LEEI*
Fiber density, % area	87.7 ± 8.3	91.0 ± 2.7	88.7 ± 1.5	82.8 ± 10.2	77.0 ± 3.0	80.5 ± 3.7

Fiber density was analyzed by quantification of the eosin-positive area fraction in hematoxylin and eosin-stained tissue sections of differently treated pericardia (Table 1). Data are presented as means ± SD (*n* = 10 randomly selected areas of three to four different animals and two to three different tissue sections each). No statistically significant differences between treatment groups were detected using Kruskal–Wallis test followed by Dunn's multiple comparisons test.

## Discussion

Pericardium-based tissue transplants encounter a limited life span due to degenerative processes and calcium deposition.^1^ The current gold standard of tissue preparation uses glutaraldehyde, which is associated with the accelerated graft failure.^11–13^ The replacement of glutaraldehyde has been suggested to lower susceptibility to calcification, promote endothelialization, and enhance tissue durability.^18^ Here, we report a novel toxin-free procedure to stabilize and sterilize pericardial tissue that combines photo-initiated UV cross-linking with LEEI.

### Decellularization efficiency

Cellular remnants in transplantable tissues have been shown to increase immunogenicity as well as favor calcification and failure of tissue-engineered heart valves.^38,39^ Thus, a decellularization protocol was applied. According to Crapo *et al.*, successful decellularization requires (1) the absence of nuclear material in hematoxylin and eosin-stained or DAPI-stained tissue sections and (2) less than 50 ng of remaining DNA per mg of extracellular matrix.^40^ The absence of nuclear material in hematoxylin and eosin-stained tissue sections as well as a 70-fold reduction in the DNA content were shown in Figure 2. The mean remaining DNA content was below the threshold defined by Crapo *et al.* Thus, the applied protocol, which was adopted from Roosens *et al.*,^29^ was effective.

### Stabilization against premature biodegradation

Digestion of transplantable tissues with collagenase is a model for the *in vivo* biodegradability after transplantation.^41^ Furthermore, it is an indirect method to estimate the cross-linking degree. A higher cross-linking degree leads to a decreased accessibility of the enzyme to cleavage sites as well as impaired penetration into deeper layers.^42^ LEEI by itself was not able to increase the tissue's resistance to collagenase. Schönfelder^34^ has even observed a dose-dependent decrease in enzymatic resistance with increasing doses using comparable parameters. Thus, various cross-linkers and radical scavengers were evaluated for the modulation of tissue stability once combined with LEEI. However, glucose, ascorbate, or riboflavin in combination with LEEI were unable to significantly influence tissue resistance to collagenase. In contrast, Ohan and Dunn found an increase in enzymatic resistance and mechanical properties of collagen films after gamma irradiation in the presence of glucose.^15^ Seto *et al.* showed a protective effect on tendons after electron irradiation in the presence of glucose and riboflavin but not with ascorbate.^14^ These discrepancies may originate from differences in tissue architecture as well as variation in experimental procedures. Nevertheless, cross-linking with UV in the presence of riboflavin and subsequent LEEI significantly decreased the pericardial digestion rate, thus significantly increasing the stability compared with decellularized pericardia. Wollensak *et al.* have shown successful cross-linking by riboflavin and UV on human corneae.^43^ Weadock *et al.* showed increased mechanical strength of bovine dermal collagen after UV irradiation.^44^

Compared with glutaraldehyde-treated pericardia, all evaluated procedures reached significantly lower enzymatic resistances. Tedder *et al.* showed that moderate cross-linking tolerates cell infiltration and gradual remodeling after subdermal implantation of porcine pericardia in rats.^45^ In accordance, Umashankar *et al.* observed remodeling potential and no apparent signs of calcification of decellularized bovine pericardia after subcutaneous implantation in rats. Glutaraldehyde-treated patches, on the contrary, did not show signs of remodeling and exhibited calcification.^46^ Additionally, biodegradability has been suggested to be essential for tissue remodeling since degraded extracellular matrix peptides are believed to act as chemoattractant for several progenitor cells *in vitro*.^47^ Thus, a moderate degradation rate could be favorable. Simultaneously, a cytocompatible surface may prevent scaffold failure due to complete degradation by infiltrating cells. Jiang *et al.* could show replacement of degraded tissue by endogenous tissue with collagen membranes after subcutaneous implantation in rats.^42^ This process could not be observed with glutaraldehyde-cross-linked tissue.^48^

### Effectiveness of irradiation and penetration depth

Sterility is a prerequisite for tissue grafts. Here, we could sterilize riboflavin/UV–LEEI-treated decellularized pericardial scaffolds applying a minimum dose of 28.7 kGy. This dose is above the dose required for sterilization by ionizing irradiation by different pharmacopoeia; thus, a sufficient SAL can be assumed. Due to the homogeneous dose distribution, not only microorganisms on the tissue surface but also within the tissue could be successfully sterilized. Despite large variation in the bioburden between batches and potential variations in the kind of microorganisms, sterility was obtained in all samples. Additionally, LEEI was shown to be an effective procedure for inactivation of viruses by Fertey *et al.*^22^ Kowalski *et al.* observed a bioburden estimate of up to 2.8 × 10^4^ CFU on human and animal tissue grafts.^49^ The bioburden determined in this study exceeds this value by one log_10_ level. This difference may originate from the high precautions taken during removal of tissue grafts compared with tissue removal for meat production.

One of the major challenges of LEEI is a limited penetration depth.^28,33^ Therefore, the maximum thickness of the pericardia that encounter no remaining viable bacteria on the tissue after LEEI was modeled by challenging different thicknesses of HDPE foil disks with >10^6^ *B. pumilus* spores in the central layer. *B. pumilus* spores serve as reference bioindicator for validation of sterilization processes using ionizing radiation because of their resistance to radiation.^35^ The dose required to reduce the viable bacterial load by 90% (one log level) is indicated as D_10_ value.^36^ For *B. pumilus* spores, a D_10_ value of 1.2 kGy was indicated by the manufacturer (MesaLabs) using a Cobald_60_ source. Using LEEI, a D_10_ value of 1.4 kGy was determined by Gotzmann *et al.*^37^ Thus, gamma irradiation and LEEI have comparable D_10_ values. Considering the D_10_ value, 8.4 kGy are theoretically required to eradicate six decimal powers of *B. pumilus* spores. Consistently, when 11.1 kGy reached the center of a 175 μm thick pericardial tissue, >10^6^ *B. pumilus* spores were inactivated. The mean thickness of all treatment groups lay below 175 μm.

For conformation of the dose calculations, a film dosimeter was placed between two pericardia and treated with LEEI. There were no significant differences between the applied and the calculated doses confirming the applied model. For thicknesses above 175 μm, the log reduction was calculated using the D_10_ value as well as the applied dose and compared with the log reduction measured by viable counts. The measured log reduction lay above the calculated log reduction confirming the expected inactivation effectiveness of *B. pumilus* spores by LEEI.

The parameters for LEEI used in this study were customized for porcine pericardial tissue. By adjusting those parameters, further tissues or scaffolds may be sterilized likewise. The penetration depth can be substantially increased by increasing the accelerating voltage from 150 to 300 kV. An accelerating voltage of 300 kV is included in the range of low-energy. Concurrently, only a minor increase in shielding requirements occurs.^33^ Furthermore, the density of freeze-dried tissues is considerably lower than the density of wet tissues. This influences the penetration depth positively. Thus, tissues and scaffolds substantially thicker than porcine pericardia may also be sterilized by LEEI after customization of the parameters used for LEEI.

### Mechanical suitability

Mechanical performance is crucial for the suitability of the pericardial scaffolds for the intended application. Unfavorable mechanical properties may lead to graft failure and reoperations. For repair of abdominal wall defects, Deeken and Lake recommend an ultimate tensile strength of at least 20 MPa and a strain at fracture between 10% and 30% using a strain rate of 25 mm/min.^50^ The mean strain at fracture of riboflavin/UV–LEEI-treated pericardia of 20.1% fits this criterion. After riboflavin/UV and LEEI treatment, the mean ultimate tensile strength lies just below the threshold recommended by Deeken and Lake. Thus, the analysis of the ultimate tensile strength during recurrent mechanical stress should be assessed to evaluate the suitability. The mean ultimate tensile strength of decellularized pericardia increased after riboflavin/UV and LEEI treatment (11.5 ± 4.2 MPa vs. 17.6 ± 8.6 MPa), but the increase was not statistically significant.

The mechanical requirements for patches for vascular reconstruction majorly depend on the site of transplantation. Miyamotto *et al.*, for example, observed an ultimate tensile strength of 19 MPa for a glutaraldehyde-treated pericardial patch for carotid enderectomy.^51^ Thus, the ultimate tensile strength of 17.6 ± 8.6 MPa determined in this study for riboflavin/UV–LEEI-treated scaffolds seems to be within a favorable range.

The mechanical deformation of a human heart valve during opening and closing is estimated to be 10%.^52^ All treated samples are within the linear region of the stress–strain curve at 10% elongation. Thus, the deformation is expected to be fully reversible. However, the stress at 10% strain of glutaraldehyde-treated tissue is significantly lower compared with riboflavin/UV–LEEI-treated pericardia. Increased tissue stress may favor calcification of bioprosthetic heart valves.^53^ Thus, it needs to be investigated whether this higher stress induces adverse events in the absence of glutaraldehyde. In accordance to Arbeiter *et al.* as well as Kayed *et al.*, we report an increase in the strain at fracture of glutaraldehyde-treated pericardia compared with native tissue.^54,55^

### Cytocompatibility

Toxicity is a major drawback associated with glutaraldehyde-treated pericardia.^13^ Due to the inability of cells to adhere and survive for longer periods on glutaraldehyde-treated tissue, formation of functional cell layers on the tissue surface as well as remodeling are not likely to occur.^48,56^ In accordance, little cell attachment and no cell proliferation was observed on glutaraldehyde-treated pericardia. To confirm that this lack of cell adhesion and proliferation is not associated with poor washing and tissue preparation, two commercially available glutaraldehyde-treated pericardial patches were evaluated. Similar results were obtained, although both tissues underwent different procedures for glutaraldehyde cross-linking and additional treatments to suppress calcification. On riboflavin/UV and LEEI scaffolds, however, no decrease in overall metabolic activity or cell number compared with decellularized pericardia was shown. After 96 h, the cell number significantly increased compared with samples analyzed after 48 h, which may indicate cell survival and proliferation on riboflavin/UV–LEEI-treated pericardial scaffolds. Thus, formation of a functional endothelial cell lining by endogenous endothelial cells *in vivo* appears feasible. Complete recellularization with endothelial cells creates an endogenous interface and establishes a nonthrombogenic surface.^57^ Moreover, xenogeneic antigens are masked and thus prevent foreign body reactions.^58^ Furthermore, endothelial cells have shown protective effects against tissue calcification.^58^ Scattered endothelial cells, as on glutaraldehyde-treated pericardia, are not sufficient to evoke those beneficial effects. In contrast, a confluent endothelial cell layer on riboflavin/UV–LEEI-treated pericardia may contribute to prolonging the tissue's durability *in vivo*.

### Tissue morphology

Tissue microstructure was analyzed by scanning electron microscopy and hematoxylin and eosin staining. Glutaraldehyde-treated samples tended to have higher fiber densities compared with all other treatment groups. This may indicate an increase in cross-linking, which is accompanied by a change in tissue morphology. The difference in fiber density between riboflavin/UV–LEEI-treated pericardia and native pericardia was smaller compared with the difference in fiber density between glutaraldehyde-treated pericardia and native pericardia. This may indicate that the tissue stabilization obtained by the SULEEI procedure, which was shown by an increase in enzymatic resistance of riboflavin/UV–LEEI-treated pericardia, is not accompanied with major morphological changes.

Roosens *et al.*, who established the decellularization procedure applied here, showed increased spaces between collagen fibers and a considerable loss in elastin and glycosaminoglycan content.^29^ A loosening of the fiber density as an effect of decellularization could also be derived as a trend from our data. It is further expected that, in accordance with Roosens *et al.*, a loss in elastin and glycosaminoglycan content in riboflavin/UV–LEEI-treated pericardia might have occurred.

### Opportunities of LEEI

Effects of ionizing irradiation on tissue morphology mainly depend on the substrate and the process parameters applied.^33^ During ionizing irradiation, the occurrence of free radicals leads to the eradication of pathogens as well as nonspecific modifications of the substrate.^20^ Fertey *et al.* and Bayer *et al.* showed that virus protein structures, for example, were not significantly altered after LEEI with an applied dose of 30 kGy. The preservation of surface protein structures was demonstrated by *in vitro* antibody binding as well as by *in vivo* formation of neutralizing antibodies in mice.^21,22^ Thus, LEEI seems nondetrimental to protein structures. In line with those findings, the presented data indicate only minor morphological changes caused by LEEI. In contrast, gamma irradiation has been reported to affect collagen structure by fibril reorganization, protein denaturation, fragmentation, and condensation.^14,25–27^ HEEI has been shown to decrease the failure load of patellar tendon allografts by applying a dose of 34 kGy.^24^ This decrease in mechanical properties could not be restored after implantation in sheep.^23^ In contrast, no impairment in the ultimate tensile strength of riboflavin/UV–LEEI-treated pericardia occurred by applying a dose of 31 kGy.

LEEI applies the required dose for sterilization of 25 kGy within several milliseconds. Thus, the exposure time to a noncontrolled ionized atmosphere is reduced to a minimum and prevents unintended tissue damage.^21,22^ Gotzmann^59^ has shown a significant influence of the exposure time on the effect of ionizing irradiation. In contrast, HEEI and gamma irradiation require several seconds and several hours, respectively, to apply equal doses.^33^ This may contribute to the tissue damage by gamma irradiation and HEEI, as described previously. Moreover, thermal effects of LEEI are negligible. Thus, heat-induced collagen degradation by LEEI is not of concern, whereas thermal effects have to be considered during HEEI and gamma irradiation.

For X-ray shielding during LEEI, 8 mm of lead were sufficient in this study. This allows the integration of an LEEI facility directly at the location of the user such as in hospitals, tissue banks, or good manufacturing practice laboratories. Thus, LEEI enables novel applications in a small and cost-effective facility. Since shielding requirements exponentially rise with increasing accelerating voltages and decreasing density of the shielding material,^60^ HEEI and gamma irradiation require heavy shielding (a few meters of concrete) and specialized companies.^33^

## Conclusion

In this study, the novel SULEEI procedure for sterilization and stabilization of decellularized pericardial scaffolds by photo-initiated UV cross-linking and LEEI was developed. Compared with glutaraldehyde-treated pericardial scaffolds and commercial pericardial patches, riboflavin/UV–LEEI-treated pericardial scaffolds showed substantially improved cytocompatibility. Additionally, the ultimate tensile strength of riboflavin/UV–LEEI-treated pericardia was maintained. Conversely, impairment in mechanical strength is of concern after HEEI and gamma irradiation of collagen-rich tissues. Thus, this may be a promising alternative procedure for processing of pericardium-based tissue transplants and potentially other thin tissue transplants. Nonetheless, further analysis including calcification analysis and *in vivo* testing is needed for complete evaluation of the suitability as pericardium-based tissue transplant. Moreover, the adjustment and implementation of the SULEEI procedure for further tissues and biomaterials showing equal thicknesses, densities, and geometries including amniotic membranes and hydrogels may possibly broaden the applications in the future.

## Supplementary Material

Supplemental data
