# Supplementary material for: Stabilization and Sterilization of Pericardial Scaffolds by Ultraviolet and Low-Energy Electron Irradiation
Source: Tissue Eng Part C Methods. 2018 Dec 1;24(12):717–29. doi: 10.1089/ten.tec.2018.0285 (PMC6306682; doi:10.1089/ten.tec.2018.0285)
Supplement: Supplemental data [file Supp_Table1.pdf]

Supplementary Data

Supplementary Table S1. Morphological Observations from Scanning Electron Micrographs and Hematoxylin and Eosin-Stained Histological Sections

Treatment	*Observation*
Native	Network of randomly oriented collagen fibers. Crimped surface structure. Small interfibrillar spaces.
Glutaraldehyde	Network of randomly oriented collagen fibers. Crimped surface structure. Small interfibrillar spaces.
dGlutaraldehyde	Separated collagen fibers. Crimped surface structure. Large interfibrillar spaces in some areas.
Decellularized	Separated collagen fibers. Partially oriented, loosely crimped fiber network. Large interfibrillar spaces.
Decellularized–LEEI	Separated collagen fibers. Highly directed, crimped fiber network. Large spaces between fiber bundles.
Riboflavin/UV–LEEI	Separated collagen fibers in some areas. Partially oriented, crimped fiber network. Large interfibrillar spaces in some areas.

Observations are based on two to five tissue sections from three to four different animals per group.
LEEI, low-energy electron irradiation; UV, ultraviolet.
